# Association between daytime napping and obesity in Chinese middle-aged and older adults

**DOI:** 10.7189/jogh.10.020804

**Published:** 2020-12

**Authors:** Nan Wang, Junyi Zou, Shu Fang, Junmin Zhou

**Affiliations:** 1 West China School of Public Health and West China Fourth Hospital, Sichuan University, Chengdu, Sichuan, China; 2 Department of Medicine, The University of Hong Kong, Hong Kong

## Abstract

**Background:**

No studies have assessed the association between daytime napping and obesity in China. The study aimed to examine the association between daytime napping and obesity among Chinese middle-aged and older adults, and to evaluate the difference between the aforementioned association in men and women.

**Methods:**

Overall, 14 685 participants aged 45 years and older were included by using data from China Health and Retirement Longitudinal Study (CHARLS) in 2015. A multivariable logistic regression model was used to investigate the relationship between daytime napping and obesity after adjusting for potential confounders. Stratified analyses were performed to examine the association differences by sex. Besides, the Cochran-Armitage test for trend was used to detect if there was a significant dose-response relationship between daytime napping and obesity.

**Results:**

The mean age of participants was 60.32, and the mean daytime napping duration was 38.97 minutes. In the sample, compared with no daytime napping group, the risks of being obese were higher in both moderate daytime napping group (1-60 minute/d) (odds ratio OR = 1.27, 95% confidence interval (CI) = 1.13-1.44) and extend long daytime napping group (>60 minute/d) (OR = 1.34, 95% CI = 1.15-1.56). In sex stratification, these significant correlations only existed in women but not in men. Compared with no daytime napping, women who napped 1-60 minute/d and over 60 minute/d were more likely to be obese (OR = 1.37, 95% CI = 1.18-1.59 and OR = 1.49, 95% CI = 1.23-1.81, respectively). Besides, the Cochran–Armitage trend test revealed that the prevalence rate of obesity increased as the daytime napping duration increased (*P* < 0.001).

**Conclusions:**

The study established the relationship between daytime napping and obesity in a general Chinese population. The association, however, was only detected among women. Furthermore, there was a dose-response relationship between daytime napping and obesity among women. Future studies may verify this association by using a longitudinal design and focus on the mechanisms behind such association.

Obesity is one of the most significant public health concerns in the world [1]. It was estimated that obesity prevalence in adults was 603.7 million in 2015 globally [2]. Obesity leads to great external costs to society, and the global costs of obesity were evaluated to be 2.8% gross domestic product or 2.0 trillion dollars in 2014 [3,4]. In China alone, because of the rapid economic growth, urbanization and changes in lifestyle, the overall obesity rate was over 20% in some areas [5]. Also, being obese is related to many health issues, such as diabetes [6], cardiovascular disease [7], several cancers [8], even reducing life quality and expectancy.

Daytime napping is a common behavior in many countries and the prevalence of daytime napping ranges from 10% to 68.6% in different groups [9-11]. It is widespread in China, too. Many Chinese regards it as a traditional and healthy lifestyle and as part of planned and regular routine across all age groups [12-14]. A large number of studies, however, have found the negative impacts of daytime napping on health outcomes. Specifically, those who napped over 1 hour/d had a 31% increased risk of developing diabetes [15]. Longer daytime napping was associated with an increased risk of hypertension [16]. A significantly positive association was examined between daytime napping and depression among 45-65 years old in both sexes [17]. Extended daytime napping was positively associated with a higher risk of stroke [18]. However, only three studies have examined the relationship between daytime napping and obesity. Two of them found daytime napping was significantly associated with obesity, while another reported insignificant association. Specifically, both of the significant studies came from the United States. One of them showed that daytime napping increased 10.4% of prevalence of obesity among participants aged 18-64 years [19], and the other one indicated daytime napping was associated with obesity in older populations (male and female participants were from two distinct cohorts) [20]. Another study in Japan found the insignificance of napping duration and obesity among the oldest population (aged ≥80 years) [21]. In addition to the inconsistent findings, the specification and categorization of napping in the above studies (eg, more than one 15-minute nap per week was regarded as napping) were not comparable to mainstream napping studies, as daytime napping was not their primary research focus. Hence, it appears to be necessary to conduct a study with an overarching goal focusing on daytime napping and obesity. Furthermore, no studies have evaluated such association in China. Thus, the primary research objective of the study was to investigate the association between daytime napping and obesity in middle-aged and older Chinese adults by using a nationally representative sample.

Besides, previous studies have shown the disparity in obesity or daytime napping in different sex groups [22-24], so the secondary objective was to examine the sex difference in the relationship between daytime napping and obesity.

## METHODS

### Study design and population

The cross-sectional data of China Health and Retirement Longitudinal Study (CHARLS) in 2015 were used. CHARLS, as an ongoing open cohort on 45 years or above of age, started in 2011, followed up with participants every 2 years, and covered 28 provinces in China. CHARLS chose multistage probability sampling to evaluate the economic, social and health status of middle-aged and elderly Chinese people. Data were collected by face-to-face computer-assisted personal interviews. Details of CHARLS can be accessed elsewhere [25].

The secondary data from CHARLS could be downloaded publicly on http://charls.pku.edu.cn/en. The total participants were under investigation with the permission of informed consent. The ethical approval of data collection was from the Biomedical Ethics Review Committee of Peking University (IRB00001052-11015) [26].

**Figure 1:**
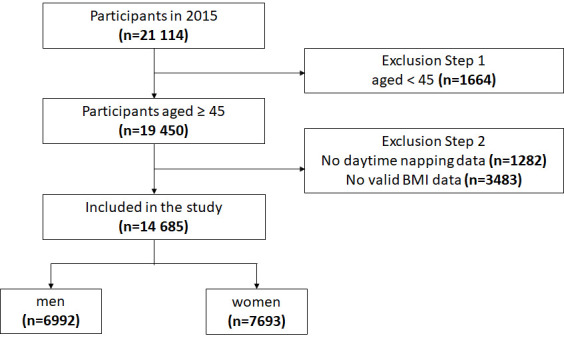
Sample included in the study.

**Figure 1** shows the data inclusion flowchart. In 2015, 21 144 participants were investigated, and the final number of the population included in this study was 14 685, including 6992 men and 7693 women. Exclusion criteria followed these steps: (1) age less than 45 years, (2) missing values of daytime napping, (3) no valid Body Mass Index (BMI) data.

To understand the possible impacts of step 2 exclusion, we compared our final sample with the sample before step 2 exclusion (ie, exclusion based on invalid data of BMI and napping) (Table S1 in the **Online Supplementary Document**).

Our final sample was not largely different from the sample before step 2 exclusion in many aspects, although the hypertension prevalence was higher in the final sample than that in the sample before exclusion (30.7% vs 23.3%). Thus, the exclusion of those samples based on invalid data of BMI and napping would not pose a grave concern.

### Measurements

#### Obesity

BMI was calculated as the weight in kilograms divided by the height in meters squared, kg/m^2^. Because of the different body shapes and skeletons among Chinese [27], the criteria for the BMI was adopted according to the National Health and Family Planning Commission of the People’s Republic of China [28]. Particularly, it has four categories: under-weight (<18.5 kg/m^2^), normal-weight (18.5-23.9 kg/m^2^), over-weight (24.0-27.9 kg/m^2^) and obesity (≥28.0 kg/m^2^). In our study, obesity was defined as BMI ≥ 28.0kg/m^2^, while non-obesity was BMI < 28.0kg/m^2^.

#### Daytime napping

Self-reported daytime napping duration was obtained by asking the question “During the past month, how long did you generally take a nap after lunch?” According to the previous literature [22,29], the outcomes on daytime napping were classified into three groups: 0 minute/d (no daytime napping), 1-60 minute/d (moderate daytime napping) and >60 minute/d (extended daytime napping).

#### Covariates

Covariates were constituted by three groups: demographic and socio-economic status, health behaviors, and health-related variables. Demographic and socio-economic status included age, community type, sex, marital status, education, annual household income and employment. Health behaviors were comprised of alcohol consumption, smoking, nighttime sleep, sleep quality and physical activity. Health-related variables included diagnosed hypertension and diabetes.

Community type was defined as “urban” (main city zone/combination zone between urban and rural areas/the town center/ZhenXiang area/special area) and “rural” (township central/village) [22]. Education was divided into “no formal education (illiterate) or did not finish primary school”, “Sishu/home school/elementary school”, and “middle school or above”. Marital status was classified into “cohabited” (married with spouse present/cohabitated) and “other” (married but not living with spouse temporarily for reasons/separated/divorced/widowed/never married). Annual household income was separated into three groups, 0 to 4000, 4001 to 25 000, and over 25 000 CNY, and the exchange rate between CNY and US dollar was 6.2 in 2015 (6.2 CNY – 1 US dollar). To complete the annual household income data, the way of linear interpolation was used to obtain all components of household income.

Alcohol consumption was divided into “yes” (drink more than once a month/drink but less than once a month) and “no”. Similarly, smoking was “yes” (current/former smokers) and “no”. Nighttime sleep was categorized by the question “during the past month, how many hours of actual sleep did you get at night (average hours for per night) (this may be shorter than the number of hours you spend in bed)”. By asking the question “my sleep was restless”, four options of “rarely or none the time (<1 day)”, “some or a little of the time (1-2 days)”, “occasionally or a moderate amount of the time (3-4 days)”, and “most or all of the time (5-7 days)” were represented as good, fair, poor and bad sleep quality, respectively. Physical activity was categorized into the binary variable as “yes” (walking or activities needing hard/high/moderate intensity physical effort for at least 10 minutes continuously during a usual week) and “no” [12,30]. Hypertension and diabetes were based on self-reported data.

### Analysis

In the descriptive analyses, mean (standard deviation, SD) and frequency (percentage) for the continuous variables and categorical variables were used, respectively. Multivariable logistic regressions were adopted to explore the association between daytime napping and obesity, after adjusting for potential confounders, including age, sex, community type, marital status, annual household income, education, employment, smoking, alcohol consumption, nighttime sleep, sleep quality, physical activity, hypertension, and diabetes. The list-wise deletion was used in the regressions for those covariates with missing values. In addition, the Cochran-Armitage test for trend was used to detect if there was a significant dose-response relationship between daytime napping and obesity. Furthermore, in order to examine the possible difference of the aforementioned association in men and women, multivariable logistic regressions were conducted separately by sex. Odds ratios (ORs) and 95% confidence intervals (CI) were reported. All statistical analyses were 2-sided, and the *P*-value less than .05 could be identified as statistical significance. SPSS for Windows (version 21.0, IBM, New York, USA) was used to complete statistical processes of descriptive and multivariable logistic regression. Trend analysis was performed by R-3.6.3.

## RESULTS

### Sample description

The descriptive details of all included variables are displayed in Table 1. The total sample had a mean age of 60.32 (SD = ±9.66). The daytime napping duration was 38.97 (SD = ±45.14) minutes (including people who don't nap). Moreover, the proportion of daytime napping were 42.2% (0 minute/d), 39.1% (1-60 minute/d), and 18.8% (>60 minute/d). The overall obesity prevalence was 12.8%.

**Table 1:** Characteristics of sample

Variable	Overall sample		Men	Women	
**Mean ± SD or frequency (%)**		**Mean ± SD or frequency (%)**	**Mean ± SD or frequency (%)**	
**Dependent variable**					
Obesity (n = 14 685)					
No	12 803 (87.2)	6289 (89.9)			6514 (84.7)
Yes	1882 (12.8)	703 (10.1)			1179 (15.3)
**Independent variables**					
**Demographic and socio-economic status**					
Age (n = 14 685):	60.32 ± 9.66	60.86 ± 9.68			59.83 ± 9.62
Community type (n = 14 647):					
Rural	10 928 (74.6)	5224 (74.8)			5704 (74.4)
Urban	3719 (25.4)	1760 (25.2)			1959 (25.6)
Sex (n = 14 685)		6992 (47.6)			7693 (52.4)
Marital status (n = 14 685):					
Other	2540 (17.3)	917 (13.1)			1623 (21.1)
Cohabited	12 145 (82.7)	6075 (86.9)			6070 (78.9)
Education (n = 13 514):					
Illiterate	6078 (45.0)	1925 (30.1)			4153 (58.3)
Primary school	3077 (22.8)	1766 (27.7)			1311 (18.4)
Middle school or above	4359 (32.3)	2695 (42.2)			1664 (23.3)
Annual household income (n = 14 685):					
0-4000	4830 (32.9)	2289 (32.7)			2541 (33.0)
4001-25000	4904 (33.4)	2340 (33.5)			2564 (33.3)
>25000	4951 (33.7)	2363 (33.8)			2588 (33.6)
Employment (n = 14 673):					
No	4647 (31.7)	1758 (25.2)			2889 (37.6)
Yes	10 026 (68.3)	5231 (74.8)			4795 (62.4)
**Health behaviors**					
Daytime napping (n = 14 685):					
0 min	6192 (42.2)	2594 (37.1)			3598 (46.8)
1-60 min	5736 (39.1)	2851 (40.8)			2885 (37.5)
>60 min	2757 (18.8)	1547 (22.1)			1210 (15.7)
Alcohol consumption (n = 14 677):					
No	9464 (64.5)	2929 (41.9)			6535 (85.0)
Yes	5213 (35.5)	4057 (58.1)			1156 (15.0)
Smoking (n = 14 666):					
No	8275 (56.4)	1268 (18.2)			7007 (91.1)
Yes	6391 (43.6)	5706 (81.8)			685 (8.9)
Nighttime sleep (hours) (n = 14 510):					
<6	4425 (30.5)	1809 (26.0)			2616 (34.6)
6-8	8676 (59.8)	4510 (64.9)			4166 (55.1)
>8	1409 (9.7)	629 (9.1)			780 (10.3)
Sleep quality (n = 14 599):					
Good	7545 (51.7)	4165 (59.8)			3380 (44.3)
Fair	2036 (13.9)	960 (13.8)			1076 (14.1)
Poor	2061 (14.1)	768 (11.0)			1293 (16.9)
Bad	2957 (20.3)	1072 (15.4)			1885 (24.7)
Physical activity (n = 14 685)					
No	8146 (55.5)	3862 (55.2)			4284 (55.7)
Yes	6539 (44.5)	3130 (44.8)			3409 (44.3)
**Health-related variables**					
Hypertension (n = 14 671):					
No	10 163 (69.3)	4923 (70.5)			5240 (68.2)
Yes	4508 (30.7)	2060 (29.5)			2448 (31.8)
Diabetes (n = 14 672):					
No	13 281 (90.5)	6408 (91.7)			6873 (89.4)
Yes	1391 (9.5)	577 (8.3)			814 (10.6)

SD – standard deviation, min – minute

In terms of sex differences, men appeared to nap more than their women counterparts (43.98% vs 34.42%), while less likely to be obese (10.1% vs 15.3%).

### Association between daytime napping and obesity

**Figure 2:**
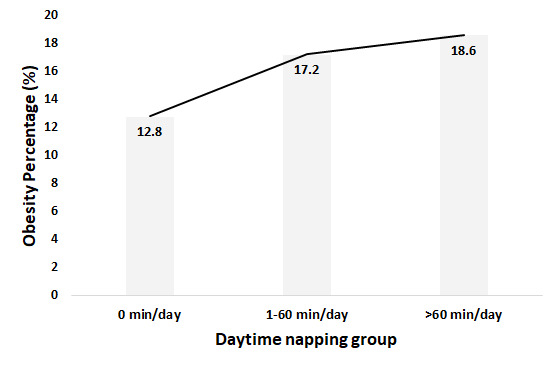
Obesity percentage by daytime napping group among women.

**Table 2** indicates the multivariable logistic regression results on daytime napping and obesity after adjusting for covariates in the overall sample, men and women. As for the overall sample, the participants who took naps showed higher odds of being obese compared with the no daytime napping counterparts. The ORs in 1-60 minute/d and >60min/d nap groups were 1.27 (95% CI = 1.13-1.44) and 1.34 (95% CI = 1.15-1.56). Stratified by sex, no significant relationship was found on daytime napping and obesity among men. However, compared to no daytime napping, the women with daytime napping had a higher probability of obesity in 1-60 minute/d group (OR = 1.37, 95% CI = 1.18-1.59) and >60min/d group (OR = 1.49, 95% CI = 1.23-1.81). In addition, the Cochran–Armitage trend test suggested that the longer women participants napped, the higher risk of obesity (*P* < 0.001). Figure 2 displayed the dose-response relationship between obesity percentage and different daytime napping group among women.

**Table 2:** Multivariable logistic regression on association between daytime napping and obesity*

Variable	Overall sample (n = 14 685)	Men (n = 6992)		Women (n = 7693)	
**Odds ratio (95% CI)**	**Odds ratio (95% CI)**		**Odds ratio (95% CI)**	
**Variables of interest**					
Daytime napping (minutes):					
0 (Ref)	1		1		1
1-60	**1.27 (1.13-1.44)**		1.10 (0.90-1.34)		**1.37 (1.18-1.59)**
>60	**1.34 (1.15-1.56)**		1.13 (0.90-1.44)		**1.49 (1.23-1.81)**
**Demographics and socioeconomic status**					
Age	**0.95 (0.94-0.96)**		**0.95 (0.94-0.96)**		**0.95 (0.94-0.96)**
Community type:					
Rural (Ref)	1		1		1
Urban	**1.30 (1.14-1.48)**		**1.40 (1.14-1.72)**		**1.24 (1.05-1.47)**
Sex:					
Men	1		-		-
Women	**1.24 (1.04-1.47)**		-		-
Marital status:					
Other (Ref)	1		1		1
Cohabited	**1.18 (1.01-1.37)**		**1.55 (1.14-2.10)**		1.07 (0.89-1.28)
Education:					
Illiterate (Ref)	1		1		1
Primary School	**0.86 (0.74-0.99)**		0.89 (0.70-1.14)		0.87 (0.72-1.05)
Middle school or above	0.92 (0.81-1.06)		1.02 (0.81-1.28)		0.87 (0.73-1.04)
Annual household income:					
0-4000 (Ref)	1		1		1
4001-25000	1.02 (0.89-1.17)		1.12 (0.90-1.40)		0.96 (0.81-1.14)
>25000	0.96 (0.84-1.11)		1.03 (0.82-1.30)		0.91 (0.77-1.09)
Employment:					
No (Ref)	1		1		1
Yes	**0.73 (0.64-0.82)**		**0.79 (0.63-0.99)**		**0.69 (0.59-0.80)**
**Health behavior**					
Alcohol consumption:					
No (Ref)	1		1		1
Yes	0.82 (0.72-0.94)		**0.84 (0.70-1.01)**		**0.76 (0.62-0.94)**
Smoking:					
No (Ref)	1		1		1
Yes	**0.82 (0.69-0.97)**		**0.75 (0.61-0.92)**		0.94 (0.73-1.21)
Nighttime sleep (hours):					
<6 (Ref)	1		1		1
6-8	1.08 (0.95-1.23)		1.08 (0.86-1.35)		1.08 (0.92-127)
>8	1.16 (0.95-1.43)		1.14 (0.80-1.62)		1.18 (0.92-1.52)
Sleep quality:					
Good (Ref)	1		1		1
Fair	0.97 (0.82-1.14)		1.04 (0.81-1.33)		0.92 (0.75-1.14)
Poor	0.94 (0.80-1.10)		0.89 (0.67-1.18)		0.96 (0.79-1.16)
Bad	**0.83 (0.71-0.98)**		0.78 (0.58-1.03)		0.85 (0.70-1.03)
Physical activity:					
No (Ref)	1		1		1
Yes	1.02 (0.92-1.13)		1.12 (0.95-1.34)		0.96 (0.83-1.10)
Health-related variables:					
Hypertension:					
No (Ref)	1		1		1
Yes	**3.15 (2.81-3.52)**		**3.34 (2.80-4.00)**		**1.25 (1.02-1.52)**
Diabetes:					
No (Ref)	1		1		1
Yes	**1.31 (1.12-1.53)**		**1.41 (1.08-1.83)**		**3.02 (2.62-3.48)**

SD – standard deviation, CI – confidence interval

*Bold values represent statistical significance.

## DISCUSSION

The study examined the association between daytime napping and obesity among men and women aged 45 years or above in China by using the cross-sectional data of CHARLS in 2015. Overall, the results suggested a significant association between daytime napping and obesity in the population. In addition, the disparity in daytime napping and obesity between sexes revealed a statistically significant association in women but not in men.

In our study, the prevalence of being obese among the overall sample was 12.8%, and it was higher in women (15.3%) than in men (10.1%). This was consistent with a previous CHARLS study conducted in 2011. The corresponding obesity rates were 11.38%, 14.28% and 8.16% in the total sample, women and men, respectively [31]. Further, the prevalence showed a slight upward trend, indicating obesity is on the rise in China, which was in line with the recent analyses [32,33]. Of all participants, 57.8% had reported daytime napping habits. Daytime napping was more prevalent in men, and the mean napping duration in women is shorter than that in men. This is in line with previous studies, which found that men were more likely to nap and longer daytime napping was more common in men than women [14,23].

Our study found a significant positive association between daytime napping and obesity. In the population of middle-aged and older Chinese, the probability of being obese was comparatively higher in those with daytime napping habits. This was partially in accordance with previous studies. As mentioned earlier, one study found daytime napping was associated with higher obesity risk, which included the population aged 18-64 [19]. In another study focusing on the oldest population (aged ≥80), however, the relationship was insignificant [21]. The disparity could be due to the different age ranges of research populations.

Further, sex disparity in daytime napping and obesity was observed. The statistically significant correlation between daytime napping and obesity only existed in women. Compared to not taking naps, women with daytime napping were associated with a higher probability of being obese. A previous study suggested that daytime napping increased the risk of being obese in both older men (OR = 1.23) and women (OR = 1.29) [20]. One possible reason for the inconsistent conclusion could be that populations in that study came from different studies and time periods, which makes the results not comparable. Furthermore, with the increase in daytime napping, the percentage of obesity in women increased. Specifically, compared with women who did not nap, the risk of obesity in extended daytime napping group (>60 minute/d) was higher than it in moderate daytime napping group (1-60 minute/d). This finding not only underscores the deteriorating effects of napping, but also suggests daytime napping as a potential target in obesity prevention and control programs in women.

The biological mechanisms underlying the link of daytime napping and obesity in women remain not fully clear. Several hypotheses could be proposed. First, the sympathetic nervous system (SNS) can be activated after stimulation by daytime napping, and SNS is positively correlated with obesity, therefore daytime napping may affect obesity status through the sympathetic nervous system [29,34]. Then, daytime napping can extend bedtime, so that the decreased thermogenesis and energy expenditure may result in obesity [35,36]. Finally, previous studies found that daytime napping and depression have a significantly positive association, and depressive symptoms are associated with decreasing estradiol levels during the transition to menopause [17,37]. Meanwhile, the significant association between menopause and obesity has been established [38]. Therefore, the menopause may have played a vital role in the relationship between daytime napping and obesity. The first two hypotheses mentioned above help to explain the biologic mechanism on the relationship between daytime napping and obesity, and the last one is useful to understand why middle-aged and older women were more likely to be obese. All the hypotheses may provide cues for exploring biological mechanisms underlying the association.

There are several limitations to the study. First, this was a cross-sectional study, the causal inference might not be concluded between daytime napping and obesity. Second, the self-reported daytime napping data might result in recall bias. Third, a large number of cases were removed in our study due to invalid data of BMI and napping. Although the exclusion of those cases did not dramatically change the profile of our samples (Table S1 in the **Online Supplementary Document**), the hypertension prevalence was higher in the final sample than that in the sample before exclusion (30.7% vs 23.3%). Caution thus should be used when interpreting our findings. Last, participants from the study were all Chinese, and it might not be representative of other groups globally. However, our study still has some strengths: (1) it was the first study to focus on the association daytime napping and obesity in middle-aged and older people in China, (2) it had a large population size and was nationally representative, (3) it found that women who napped were at a higher risk of being obese, which may provide evidence to guide future research.

## CONCLUSIONS

The findings in the study revealed the significant association between daytime napping and obesity. This association, however, only existed in middle-aged and older women. Future studies may verify this association by using a longitudinal design and focus on the mechanisms behind such association.

## Additional material

Online Supplementary Document
