# Supplementary material for: Association between daytime napping and obesity in Chinese middle-aged and older adults
Source: J Glob Health. 2020 Dec 6;10(2):020804. doi: 10.7189/jogh.10.020804 (PMC7719355; doi:10.7189/jogh.10.020804)
Supplement: Online Supplementary Document [file jogh-10-020804-s001.pdf]

Table S1. Comparison of the final sample after exclusion with the sample before exclusion based on invalid data of BMI and napping[†]

Variable	Final sample after exclusion		Sample before exclusion based on invalid data of BMI and napping	
	Number	Mean/Percentage (SD)	Number	Mean/Percentage (SD)
**Demographic and Socio-economic status**				
**Age**	14 685	60.32±9.66	4765	60.17±11.54
**Community type (n=14 647)**			**Community type (n=4730)**	
Rural	10 928	74.6	3112	65.8
Urban	3719	25.4	1618	34.2
**Sex (n=14 685)**			**Sex (n=4765)**	
Men	6992	47.6	2499	52.4
Women	7693	52.4	2266	47.6
**Marital status (n=14 685)**			**Marital status (n=4762)**	
Other	2540	17.3	1188	24.9
Cohabited	12 145	82.7	3574	75.1
**Education (n=13 514)**			**Education (n=4278)**	
Illiterate	6078	45.0	1717	40.1
Primary School	3077	22.8	824	19.3
Middle school or above	4359	32.3	1737	40.6
**Annual household income (n=14 685)**			**Annual household income (n=4765)**	
0-4000	4830	32.9	1201	25.2
4001-25000	4904	33.4	1482	31.1
>25000	4951	33.7	2082	13.7
**Employment (n=14 673)**			**Employment (n=4602)**	
No	4647	31.7	2740	59.5
Yes	10 026	68.3	1862	40.5
**Health behaviors**				
**Alcohol consumption (n=14 677)**			**Alcohol consumption (n=4638)**	
No	9464	64.5	2933	63.2
Yes	5213	35.5	1705	36.8
**Smoking (n=14 666)**			**Smoking (n=4655)**	
No	8275	56.4	2482	53.3
Yes	6391	43.6	2173	46.7
**Nighttime sleep (n=14 510)**			**Nighttime sleep (n=3512)**	
<6h	4425	30.5	1059	30.2
6-8h	8676	59.8	2130	60.6
>8h	1409	9.7	323	9.2
**Sleep quality (n=14 599)**			**Sleep quality (n=3534)**	
Good	7545	51.7	1874	53.0
Fair	2036	13.9	487	13.8

Variable	Final sample after exclusion		Sample before exclusion based on invalid data of BMI and napping	
	**Number**	**Mean/Percentage (SD)**	**Number**	**Mean/Percentage (SD)**
Poor	2061	14.1	459	13.0
Bad	2957	20.3	714	20.2
**Physical activity (n=14 685)**			**Physical activity (n=4765)**	
No	8146	55.5	3216	67.5
Yes	6539	44.5	1549	32.5
**Health-related variables**				
**Hypertension (n=14 671)**			**Hypertension (n=3771)**	
No	10 163	69.3	2845	75.4
Yes	4508	30.7	926	24.6
**Diabetes (n=14 672)**			**Diabetes (n=4636)**	
No	13 281	90.5	4195	60.5
Yes	1391	9.5	441	9.5

† Abbreviations: SD, standard deviation; min, minute; h, hour
